# Changes in Gut Microbiota and Multiple Sclerosis: A Systematic Review

**DOI:** 10.3390/ijerph20054624

**Published:** 2023-03-06

**Authors:** Alba Ordoñez-Rodriguez, Pablo Roman, Lola Rueda-Ruzafa, Ana Campos-Rios, Diana Cardona

**Affiliations:** 1 The Virgen de la Arrixaca Hospital, 30120 Murcia, Spain; 2 Faculty of Health Sciences, Department of Nursing, Physiotherapy and Medicine, University of Almeria, 04120 Almeria, Spain; 3 Health Research Center, University of Almería, 04120 Almeria, Spain; 4 Laboratory of Neuroscience, CINBIO, University of Vigo, 36310 Vigo, Spain; 5 Laboratory of Neuroscience, Galicia Sur Health Research Institute (IISGS), 15706 Vigo, Spain

**Keywords:** multiple sclerosis, gut microbiota, microbiome: short-chain fatty acid

## Abstract

Introduction: Multiple sclerosis (MS) is a chronic inflammatory neurodegenerative disease mediated by autoimmune reactions against myelin proteins and gangliosides in the grey and white matter of the brain and spinal cord. It is considered one of the most common neurological diseases of non-traumatic origin in young people, especially in women. Recent studies point to a possible association between MS and gut microbiota. Intestinal dysbiosis has been observed, as well as an alteration of short-chain fatty acid-producing bacteria, although clinical data remain scarce and inconclusive. Objective: To conduct a systematic review on the relationship between gut microbiota and multiple sclerosis. Method: The systematic review was conducted in the first quarter of 2022. The articles included were selected and compiled from different electronic databases: PubMed, Scopus, ScienceDirect, Proquest, Cochrane, and CINAHL. The keywords used in the search were: “multiple sclerosis”, “gut microbiota”, and “microbiome”. Results: 12 articles were selected for the systematic review. Among the studies that analysed alpha and beta diversity, only three found significant differences with respect to the control. In terms of taxonomy, the data are contradictory, but confirm an alteration of the microbiota marked by a decrease in Firmicutes, Lachnospiraceae, *Bifidobacterium*, *Roseburia*, *Coprococcus*, *Butyricicoccus*, *Lachnospira*, *Dorea*, *Faecalibacterium*, and *Prevotella* and an increase in Bacteroidetes, *Akkermansia*, *Blautia*, and *Ruminocococcus*. As for short-chain fatty acids, in general, a decrease in short-chain fatty acids, in particular butyrate, was observed. Conclusions: Gut microbiota dysbiosis was found in multiple sclerosis patients compared to controls. Most of the altered bacteria are short-chain fatty acid (SCFA)-producing, which could explain the chronic inflammation that characterises this disease. Therefore, future studies should consider the characterisation and manipulation of the multiple sclerosis-associated microbiome as a focus of both diagnostic and therapeutic strategies.

## 1. Introduction

Multiple sclerosis (MS) is a chronic, inflammatory, neurodegenerative condition caused by autoimmune reactions which progressively demyelinate the central nervous system (CNS) and the spinal cord [1,2]. It seems to begin when autoreactive T cells cross the blood–brain barrier (BBB) and provoke specific cascades in the CNS, leading to inflammation and axonal degeneration [3,4], although it is not clear what causes T cells activation [5].

MS affects an estimated 2.3 million people worldwide and its incidence is increasing from 50 to 300 per 100,000 inhabitants, affecting women 3-fold times [1,6,7]. It is the most common non-traumatic neurological disabling disorder in young people. It causes disability, including intestinal disfunction in more than 70% of cases [8], cognitive impairment, and a severe decrease in quality of life in young adults between 20 and 40 years old [7,9]. MS aetiology remains unclear; interactions between environmental and genetic factors appear to promote the disease [2,6,9,10,11]. In addition to genetics, environmental factors such as obesity, tobacco use, microbiota alterations, Epstein–Barr virus (EBV) infection or vitamin B deficiency play an important role in progression of the disease [10,12]. Regarding the evolution of the disease, MS has been classified into subgroups such as relapsing–remitting (RRMS), secondary progressive (SPMS), primary progressive (PPMS), progressive–relapsing (PRMS), and benign (BMS) [13]. In RRMS (83–90% of cases), the flare-ups of neurological symptoms are practically reversible, which recur unpredictably and may disappear completely or leave some sequelae, and, between relapses there seems to be no progression of MS. SPMS is described as a disease of continuous progression, with or without flare-ups, irrelevant remissions, and phases of stability. Only 10% of patients present with PPMS, which starts with disabling flare-ups with no response to treatment and has a slow onset and progressive deterioration. RPMS is characterized by occasional exacerbations in a progressive course of the disease. Finally, in BMS, after the diagnosis of the disease, the patient retains functional capacity for 10–15 years [14].

Research is currently focusing on the influence of the gut microbiota (GM) on the onset and development of MS [15,16,17]. GM is the combination of bacteria, fungi, archaea, eukaryotes, and viruses that reside in the intestinal mucosa, and Actinobacterium, Bacteroidetes, Firmicutes, Fusobacteria, Proteobacteria, and Verrucomicrobia are the main phyla that compose it [18]. GM microorganisms contribute to food digestion and fermentation, nutrient absorption, vitamin synthesis, epithelial cell maturation, intestinal barrier integrity, protection against inflammation and pathogens, and metabolic regulation [19,20].

GM may impact on the CNS and participate in its regulation through neurochemical changes, while the CNS is a crucial element in the regulation of gut function and homeostasis. This complex interaction is well-known as the gut–brain axis (GBA) [21]. Bidirectional interactions between gut and brain have an important role in gastrointestinal function modulation such as motility, secretion, blood flux regulation, intestinal permeability, immunity activity, and visceral sensations, including pain, where evidence suggests that GM has a vital role. GM can interact with the brain through activation of immune, endocrine, and neural pathways, including vagal afferents and through microbial metabolites which act directly or indirectly in the brain [22,23,24].

Some molecules derived from microorganisms, such as short-chain fatty acids (SCFAs), may have a relevant role in the gut-brain axis. SCFAs such as butyric acid (BA), acetic acid (AA), and propionic acid (PA) are produced in the colon by non-digestible carbohydrates undergoing bacterial fermentation [21,25]. SCFAs have important immunomodulatory functions mediated by increasing the number of T regulatory cells and suppressing the collaborative T cells (Th) 17 and 1, which lead to an anti-inflammatory response state [26]. Likewise, SCFAs can cross the BBB by using transporters located in the endothelial cells and influence CNS neuroinflammation [27,28]. Specifically, BA, compared to PA and AA, has strong immunomodulatory properties and regulates inflammatory processes by maintaining the balance of Th 17 cells and the levels pro and anti-inflammatory cytokines [25,29].

A disruption in GM composition, so-called gut dysbiosis, plays a fundamental role in several autoimmune conditions, including intestinal inflammatory disease, rheumatoid arthritis, and type 1 diabetes [30]. MS has also been associated with dysbiosis, including depletion and enrichment of certain bacteria in patients compared to healthy people [31,32,33]. However, a cause–effect relationship between MS and intestinal dysbiosis has not been clearly established. Considering all of the above, there seems to be an association between MS and GM. Thus, the objective of the present work is to perform a systemic review about the relation between intestinal microbiota and MS.

## 2. Materials and Methods

The systematic review was conducted in the first trimester of 2022 using studies published between January 2018 and March 2022. The PRISMA (Preferred Reporting Items for Systematic reviews and Meta-Analyses) recommendations were utilized [34].

### 2.1. Databases

The PICO (Patient, Intervention, Comparation, Outcome) method was used to design a search strategy. Accordingly, the objective of review was reflected in the question: “does it exist a relation between intestinal microbiota and MS?”

Articles were selected and collected from 6 electronic databases: PubMed, Cochrane Library, ProQuest, The Cumulative Index of Nursing and Allied Literature Complete (CINAHL), ScienceDirect, and Scopus. The terms used to access to the articles of interest in the mentioned databases were a combination of natural language and structured language using the Medical Subject Heading (MeSH) thesaurus: “multiple sclerosis”, “gut microbiota”, and “microbiome”, and using “AND” between terms and “OR” between synonyms. Research strategies are shown in Table 1.

### 2.2. Study Eligibility Criteria

The inclusion criteria used for this review were (i) Cohort studies, transversal studies, patient and control comparative studies, and comparative cohort studies (analytic observational studies) in MS patients, (ii) articles analysing GM in MS patients, (iii) articles analysing SCFAs in intestinal metabolome, (iv) articles including a population of study composed of MS diagnosed individuals between 18 and 70 years old (including all the MS subtypes) and (v) studies published in both English and Spanish.

In addition, the exclusion criteria included (i) systematic reviews, metanalysis, book chapters, doctoral dissertations, end-of-study projects, congress publications, clinical protocols, and letters to the editor, (ii) other study designs, such as interventional studies and studies without a control group, and (iii) articles analysing intestinal metabolome, but no SCFAs.

Restrictions in relation to geographical location, setting (community or hospital), or the course of the clinical study were not applied.

### 2.3. Selection of Studies and Methodological Quality

Study eligibility was performed in three phases. The first phase consisted of reading the title of identified articles in the research database. Once selected, all abstracts were reviewed in a second phase, and, finally, a full reading was used to clarify the suitability of the article for analysis. The eligibility process was conducted by the first two authors (AOR, PR) independently and in duplicate; if consensus could not be achieved, a third author (DC) was consulted. In relation with the included studies, a bibliometric analysis was performed on the following variables: (i) author and year, (ii) number of participants and controls, (iii) intestinal microbiota changes compared to control group, and (iv) changes in SCFAs compared to control groups.

Regarding the quality of the studies, the Newcastle Ottawa scale (NOS) [35], which evaluates bias in observational studies, was applied. The NOS records 8 items with 3 subscales and is scored up to 9 points. A study is considered to be of high quality when its score is ≥7. It uses predefined criteria and assigns up to 9 stars, with a maximum of 4 stars for the quality of patients selected, 2 for the comparability between cases and controls, and 3 starts for exposure or outcomes.

## 3. Results

**Figure 1:**
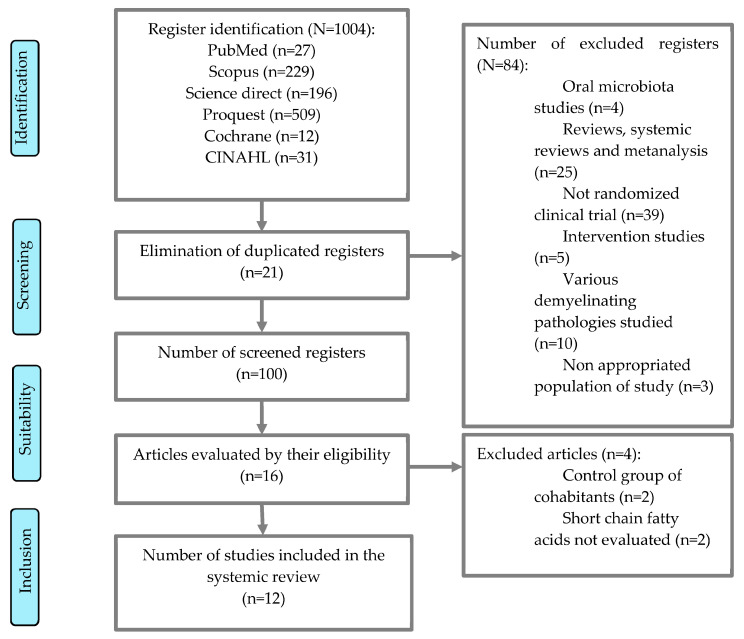
Flowchart.

After the database research, 1004 results were obtained (27 in PubMed, 229 in Scopus, 196 in ScienceDirect, 509 in Proquest, 12 in Cochrane, and 31 in CINAHL). Articles related to the objective, which fulfilled the inclusion criteria, were selected, and duplicated articles were discarded. Preliminary title selection facilitated the exclusion of duplicated articles (21 articles excluded after reading title and abstract). After selection, 100 results were obtained. Subsequently, articles that were not relevant to the topic (oral microbiota), interventional articles, animal studies, or studies on other demyelinating pathologies were excluded, resulting in 16 articles. The next step consisted of a second reading of the full text based on the exhaustive analysis of the study, excluding all the articles which did not fit because of inadequate participants. Finally, 12 articles were obtained for final revision, as shown in the flowchart (Figure 1).

### 3.1. Studies Characteristics

Twelve articles were included in this research [17,36,37,38,39,40,41,42,43,44,45,46]. The characteristics of the studies reviewed, as well as the main variables analysed, are listed in Table 2. All studies involved a total of 570 MS cases and 478 controls, i.e., healthy subjects without a diagnosis of the disease. A total of 54% of the study population was diagnosed with MS. The majority (301/570, 53%) of the cases presented a remittent–recurrent course, while 9.3% (53/570) were diagnosed with PPMS, and 3.5% (20/570) were diagnosed with BMS. A total of 34.4% (196/570) were diagnosed with MS without subtype specification. Seven studies used McDonald 2010 criteria for MS diagnosis, one used Poser criteria [44], and four did not specify the diagnosis method [37,39,41,45].

All the studies provided demographic data, with the women/men ratio being 388/182 (68%/32%) for MS and 394/184 (61.5%/38.5%) for controls. Only in one study was the percentage of men higher, 60% of MS cases and 53% of controls [40]. Furthermore, two studies reported on the ethnicity of patient, one of them distinguishing between Caucasian, Hispanics, and Afroamericans [38], and another only identified Caucasians, where 80% of the controls were Caucasian compared 95% of the MS cases [17]. Three studies recruited the participants in the USA [17,38,39], two in Spain [36,45], one in Italy [37], one in Belgium [46], and one in China [42], Brasil [44], Israel [41], Egypt [43] and Russia [40]. As shown in Table 3, all the revised articles were low risk regarding NOS scale [35].

### 3.2. Microbial Dysbiosis

Ten of the twelve selected articles evaluated GM, and eighty percent analysed alpha and beta diversity. Alpha diversity was evaluated in eight studies. On the one hand, a decrease in alpha diversity was observed in RRMS cases [39], while an increase in alpha diversity was shown in PPMS [40]. In the remaining studies, no statistically significant differences were found, affirming that there are no apparent discrepancies in the diversities between MS cases and controls.

### 3.3. Taxonomy Diversity

Analysing the specific taxonomic differences in the assessed articles, we found no uniform observations among the studies as shown in Table 4 [36,38], whereas it diminished in 40% of the studies [17,37,42,46]. At the phylum level, Firmicutes was observed to increase in 20% of the studies [36,38], while, conversely, it decreased in 40% of them [17,37,42,46]. Bacteroidetes increased in 30% [39,43,44] and diminished in 10% of the cases [17]. Actinobacteria increased [36] and decreased in 10% of the studies [44]. Proteobacteria and Lentispharaea decreased in 10% of the studies [36].

Regarding the class, Clostridia increased in one study [38] and decreased in another one [40]. With respect to families, the family Lachnospiraeae significantly decreased in controls, and Ruminococcaceae increased [40] and decreased in controls [44]. Furthermore, two articles found a decrease in bacteria of the genus *Bifidobacterium* [17,44]. Similarly, two other articles found a decrease in *Coprococcus* [37,39], *Butyricoccus* [42,46], and *Lachnospira* [37,38]. In contrast, *Akkermansia* was significantly increased compared to controls [37,38,40]. Blautia was also found increased in three articles [36,42,44] but decreased in two other works [37,38]. There was also controversy regarding *Parabacteroides*, which was increased in two studies [44,46], but decreased in two others [17,37]. This same divergence was observed in other genera, such as Dorea, which was both augmented [38] and decreased in MS depending on the research [37,42]. Likewise, *Ruminococcus*, *Faecalibacterium*, *Prevotella*, *Methanobrevibacter*, and *Dialister* also increased and diminished depending on the article [17,36,37,38,42,46].

Two studies evaluated GM at different phases of MS. Stratifying MS patients according to disease severity showed significantly less diversity in SPMS compared to RRMS and controls [37]. Other studies compared intestinal microbiota in different MS subtypes, considering the use of interferon. Microbiota richness was lower in RRMS patients treated with interferon and patients with non-treated RRMS during the relapse compared to BMS and PPMS. Controls and non-treated active RRMS showed an intermediate microbial richness [46].

In this regard, the 10 revised articles agree that MS patients have a different intestinal microbiota than controls, with different abundancies depending on the microbiota [17,36,37,38,39,40,42,43,44,46].

### 3.4. Metabolome

Four of the twelve studies analysed SCFA levels in intestinal metabolome, and those levels were compared between patients and controls [17,37,41,45]. These four selected articles analysed serum SCFA levels, finding decreases in BA [37,41] and increases in AA [45]. Consistent with this, there was a trend towards a decrease in the SCFAs in the faeces of MS patients compared to controls [17].

## 4. Discussion

The causes of multiple sclerosis are unknown, but there is evidence to indicate that GM may influence the immune system and, consequently, impact on the disease. Therefore, our aim was to analyse recent literature with the objective of investigating the relation between intestinal microbiota and MS. In the present systematic review, 12 case-control studies with intestinal dysbiosis were included, as well as SCFA alterations in patients with MS.

### 4.1. Microbial Dysbiosis

As for studies examining alpha diversity and beta diversity, only three studies found significant differences in MS compared to the controls. A decrease in alpha diversity was found in RRMS associated with cases of chronic low-grade inflammation [39,47]. This diversity was observed in other autoimmune diseases, such as inflammatory bowel disease [48,49], preclinic type 1 diabetes [50,51], and psoriatic arthritis [52], as well as inflammatory diseases such as obesity [53]. Previous studies also indicate that alpha diversity tends to decrease in patients with normalized active RRMS during remission [54]. An increase in alpha diversity [40] related to PPMS was also found. This MS subtype is quite strange [14], so the information about the structure and composition of intestinal microbiota is scarce. These changes in alpha diversity can be explained depending on whether the disease is active or not. In addition, it is necessary to elucidate whether these changes are the product of an immune response or whether they promote autoimmunity. In this regard, recent research proposes that EBV infection contributes to the production of B cells that stimulate the activation of these CNS inflammatory responses [55].

On the other hand, changes in beta diversity were observed, with no changes in alpha diversity between Hispanic American subjects with MS and controls [38]. Furthermore, a difference in beta diversity was also found in other previous studies [32,54,56,57]. According to our results, other reviews found no significant differences between alpha and beta diversity in MS [58,59].

### 4.2. Bacterial Taxonomy

Taxonomic differences reflected in the revised studies are quite diverse, making it difficult to draw firm conclusions. For this reason, and to simplify our results, we will focus on highlighting differences in gut microbial communities between MS cases and matched controls in two or more studies. Thus, we observed a decrease in Firmicutes phylum [17,42,46] and an increase in phylum Bacteroidetes [39,43]. These phyla are SCFA producers with immunoregulatory functions and, therefore, their alterations affect MS [60]. These alterations have also been detected in Chron’s disease [61].

Regarding intestinal bacteria families, Lachnospiraceae was found diminished [17]. Previous studies have shown a decrease in this family in MS patients [54], a decrease that was also observed in Alzheimer patients [62].

Intestinal bacteria genres *Bifidobacterium* [17,44], *Roseburia* [37,42], *Coprococcus* [37,39], *Butyricicoccus* [42,46], *Lachnospira* [17,37], *Dorea* [37,42], *Faecalibacterium* [17,42], and Prevotella [17,38] were also found to be decreased. Bifidobacterium has a fundamental role in immune response regulation as well as SCFA production, specifically AA [63]. Previous data corroborate the findings of this review, as low levels of this bacterium have been linked to MS [64]. In fact, probiotic administration might produce an anti-inflammatory effects in MS patients [65].

*Prevotella*, which is associated with a fibre-rich diet and has regulatory functions through the generation of butyrate [54], also decreased. This decrement has been observed in previous studies [32,57,66,67], which support a possible link between this bacteria and MS, as is the case with other conditions such as diabetes mellitus type 2 [68] or non-alcoholic fatty liver [69].

The *Faecalibacterium* low levels are consistent with the levels observed in other studies [57,70,71,72], as well as in other diseases such as inflammatory intestinal conditions and irritable bowel syndrome [61,73,74]. *Faecalibacterium* can convert acetate and lactate into butyrate [32], so these bacteria are considered butyrate producers. Butyrate is thus reduced in inflammatory conditions such as MS [75]. Among its properties, its capacity to attenuate inflammation has been shown in preclinical studies of colitis in mice by modulating mucosa T cells [76].

Similarly, *Coprococcus*, *Butyricicoccus*, and *Lachnospira*, butyrate-producing bacteria, have been observed diminished in previous studies and in other pathologies [56,67,72,77,78]. *Roseburia*, also decreased in MS patients, is a SCFA producer and essentially a butyrate producer [79]. In addition, *Roseburia* reduction has been observed in other pathologies such as juvenile idiopathic arthritis [80,81], Behcet syndrome [66,82], irritable bowel syndrome, obesity, type 2 diabetes, nervous system affections, and allergies [79,83,84,85].

Although some of the revised studies in the present work have found a decrease in *Dorea*, other research has shown an increase in MS patients [86] and also in other pathologies such as Chron’s disease [87]. Therefore, *Dorea* appears to have either proinflammatory or anti-inflammatory functions depending on the surrounding intestinal bacteria and/or the available nutrients [86].

In contrast, an increase in *Akkermansia* [37,38,40], *Blautia* [36,42,44], and *Ruminococcus* [36,46] has been demonstrated. *Akkermansia* has immunoregulatory effects by converting mucin to SCFAs [26]. However, as it degrades intestinal mucosa, it can cause intestinal inflammation [88]. Its increase has also been observed in previous studies [32,54,56,57,72] supporting a possible link between its increase and MS, as occurs in other conditions such as Parkinson’s [89,90] and in children with autism spectrum disorders (ASD) [91]. Regarding *Blautia*, it is an acetate producer [92], which can impulse insulin release and promote metabolic syndromes such as hyperglyceridemia, fatty liver disease, and insulin resistance [93]. *Ruminococcus* plays an important role in SCFA production and in decreasing inflammation. Furthermore, it is part of the healthy GM, although some species degrade mucosa and, consequently, increase inflammatory conditions such as in MS [94].

It is worth noting that GM composition is subjected to many complex interactions and there are many confounding factors that might influence its healthy levels, making a comprehensive comparison difficult. The disparity in GM among revised studies might be related to patient and control characteristics: MS types, age, disease duration, sample ubication, ethnicity, and the intake of disease-modifying drugs [95,96,97,98]. How these factors contribute to variation in GM is complex, context-dependent, and not completely understood [97].

Accordingly, differences in study methodology could explain, at least in part, the variability observed among studies. Thus, revised studies have used different protocols regarding stool collection, as some were collected by the participants themselves at home [37,38,39,43,44,45,46], while others were collected at the hospital [17,36,40,42]. Differences were also observed in the amplification of the V region of the 16S rRNA target gene, with some studies using the V3-V4 region [37,40,42,44], and others only amplifying the V4 region [36,38,39,46].

### 4.3. SCFA Alteration

Regarding SFCAs, revised articles indicated a decrease in BA serum levels in MS patients, in agreement with a decrease in SCFA-producing bacteria [37,41]. These results are consistent with previous studies showing a decrease in many butyrate-producing bacteria in MS patients [32,37,72], as well as in other autoimmune diseases [50,52]. Similarly, an increase in AA was found in MS patients [45]. This is the most abundant SCFA produced by intestinal bacteria, although it may also be converted to acetyl-CoA by glycolysis. Furthermore, some colonic bacterial strains can convert butyrate through cross-feeding mechanisms [27]. Under conditions of intestinal dysbiosis, SCFA production is often reduced, contributing to an inflammatory environment [99]. In animal models, some results suggest that SCFAs influence the pathogenesis of experimental autoimmune encephalomyelitis and, consequently, the same context is likely to be found in MS [100]. SCFAs produced by GM may alter cellular activity, contribute to modulated immune cells [101], and may also have inhibitory effects on EBV reactivation in MS [55]. Therefore, future research should evaluate the role of GM and EBV reactivity in MS.

In fact, in recent years, accumulated evidence on the protective effect of SCFAs has been updated in preclinical data and, recently, in MS patients. Studies support the possibility that SCFAs are potential bidirectional regulators [102]. Several studies have confirmed that SCFAs can promote T cell differentiation directly into proinflammatory cytokine-producing T cells depending on the cytokine context. Thus, SCFAs and their receptors may have the potential to regulate CNS autoimmune inflammation both positively and negatively [103,104]. In particular, SCFAs can cross the BBB via endothelium-localised transporters [27,28]. Therefore, in a dysbiotic situation, the production of SCFAs decreases, which would contribute to an inflammatory state favouring neuroinflammation [99].

It is worth mentioning that if a high fibre diet is ingested, SCFA levels might be drastically altered [105], and it has been suggested that such diets are related to increased levels of butyrate production [106]. Of the four revised studies, only one indicates a dietary control, finding a negative correlation between meat intake and levels of SCFA-producing bacteria [17]. In addition to diet, other factors that alter SCFA levels, such as body mass index, smoking, drug treatment, and probiotic intake [107,108,109,110,111], could be taken into account in future research. There are only few studies evaluating the effect of disease-specific drugs on GM, although some studies suggest that these therapies may restore the intestinal ecosystem to a state of eubiosis [112]. Our results seem to point in this line, as interferon beta-treated patients have similar bacterial abundancy to heathy subjects in different taxa, which are altered in untreated MS patients [36].

In terms of GM modulation, probiotic intake has been found to improve mental health in MS patients, possibly by reducing levels of inflammatory and oxidative biomarkers and decreasing insulin resistance [113,114]. In fact, preclinical studies suggest that probiotic intake may have beneficial effects in reducing the incidence and severity of MS, delaying its progression, and ameliorating motor function impairment. These effects might be mediated by the modulation of immune and inflammatory markers and the GM composition [115].

Ultimately, the studies reviewed in this article highlight the relationship between GM and MS, although a cause–effect relationship between MS and dysbiosis has not yet been established [94,116]. However, new research suggests that disturbed GM may lead to deficient SCFA production by intestinal bacteria, which may deplete the beneficial anti-inflammatory effects on the CNS [117]. Therefore, future work might consider the characterisation and modulation of the MS-associated microbiota as a strategic diagnostic and therapeutic target.

### 4.4. Limitations

In the present review, several limitations have to be determined. The main restriction is that methodological differences between the revised studies are not considered. In addition, all the included studies used a relatively moderate sample size, with a total of 570 MS cases and 478 controls from different regions of the world. Finally, we have reported the results in the taxon observed similarly in two or three studies; some associations might be overlooked, especially in the least abundant taxon.

## 5. Conclusions

Despite a modest cohort size, diversity in geographical location of participants, and sample processing, the present systemic review brings to light a dysbiosis of the GM in MS patients compared to healthy controls. More specifically, and despite variability among different studies, consistent patterns have been found, as many taxa were identified as over- or under-represented in MS compared to controls. Most of the altered bacteria are SCFA producers, which might explain the chronic inflammation which characterises this disease. Therefore, future research should consider the characterisation and modulation of the MS-associated microbiota as a target for diagnosis and therapy.

## Figures and Tables

**Table 1:** Description of the research strategies performed in each database.

Database	Search Strategy
PubMed	((((microbiome or gut microbiota [Title/Abstract]) AND Clinical Trial[ptyp])) AND ((((multiple sclerosis [Title/Abstract])) AND Clinical Trial[ptyp]))
Cochrane	“gut microbiota” or microbiome and “multiple sclerosis”
ProQuest	“gut microbiota” or microbiome and “multiple sclerosis”
CINAHL	“gut microbiota” or microbiome and “multiple sclerosis”
ScienceDirect	“gut microbiota” or microbiome and “multiple sclerosis”
Scopus	(TITLE-ABS-KEY (“gut microbiota”) OR TITLE-ABS-KEY (microbiome) AND TITLE-ABS-KEY (“multiple sclerosis”)) AND PUBYEAR > 2017 AND (LIMIT-TO (DOCTYPE, “ar”))

**Table 2:** Summary of the articles included in the study.

Reference	Participants Cases/Control ♂/♀ ♂/♀	Microbial Dysbiosis and SCFAs Metabolome
[17]	24 RRMS/25 3/21 3/22	No ≠ diversity α
		↓ *Clostridium leptum* and *Bacteroides thetaiotaomicron*
		↓ *Faecalibacterium*, *Prevotella*, *Lachnospiraceae anaerostipias*, *Bifidobacterium longum*, *Faecalibacterium prausnitzii*, *Parabacteroides* and *Escherichia*
		↓ +SCFAs
[36]	30 RRMS/14 9/21 7/7	No ≠ diversity α
		↑ Firmicutes and Actinobacteria
		↓ Proteobacteria and Lentisphaerae
[43]	30 RRMS/20 13/17 8/12	↑ *Bacteroides fragilis*
		↑ *Bacteroide fragilis* with 30 years old
		↑ *Bacteroides* relapse rate ≥ 1.4
[41]	129 MS/58 36/93 29/29	↓ butyrate
[44]	18 RRMS/18 2/16 2/16	No ≠ diversity α
		↑ Bacteroides and ↓ Actinobacteria
		↑ *Bacteroides*, *Flavobacterium* and *Parabacteroides*
		↓ *Bifidobacteria* and *Streptococcus*
[42]	22 MS/33 8/14 12/21	No ≠ diversity α
		↓ *Faecalibacterium*, *Roseburia*, *Haemophilus*, *Bilophila*, *Dorea*, *Butyricicoccus*, *Gemella*, *Clostridium* XIVb and *Granulicatella*
[45]	95 RRMS/54 30/65 21/33	↑ plasmatic acetate
		No ≠ propionate nor butyrate
[37]	26 RRMS 12 SPMS/38 18/20 18/20	No ≠ diversity α
		↓ *Lachnospiraceae*
		↑ *Akkermansia*, *Collinsella*, *Eubacterium* and *Prevotella*
		↓ *Parabacteroides*, *Roseburia*, *Coprococcus* and *Blautia*
[39]	26 RRMS/39 4/22 12/27	↓ diversity α
		↑ Bacteroidetes
		↓ *Coprococo, Clostridium*, nc. *Ruminococcaceae*, *Paraprevotella and Methanobrevibacter*
[40]	15 PPMS/15 9/6 8/7	↑ diversity α
		↑ Verrucomicrobia
		↑ *Actinomycetaceae*, *Verrucomicrobiaceae*, *Desulfovibrionaceae*, nc. *Firmicutes*, *Acidaminococcaceae*, nc. *Clostridia*, *Eubacteriaceae*, *Verrucomicrobiaceae*, *Oxalobacteraceae*, *Christensenellaceae* and *Corynebacteriaceae*
		↑ *Gemmiger* and nc. *Ruminococcaceae*
[38]	45 MS/44 15AC 16AH 14AA 11/34 16/28	MS vs. control: ↑ *Clostridia*
		MS AC vs. control: ↑ Verrucomicrobia and ↑ Akkermansia
		MS AH and AA vs. control: ↑ *Adlercreutzia*
		MS AH vs. control: ↑ *Blautia*, *Holdemanía* and *Dorea* ↓ *Prevotella*, *Slackia*, *Lachnospira* and *Dialister*
		MS AA vs. control: ↑ *Butyricococcus*
[46]	98 (52 RRMS o 26 PPMS o 20 BMS)/120 39/59 48/72	No ≠ diversity
		↑ *Alistipes*, *Anaerotruncus*, *Clostridium cluster IV*, *Lactobacillus*, *Methanobrevibacter*, *Olsenella*, *Parabacteroides*, *Ruminococcus*, *Sporobacter*
		↓ *Butyricicoccus*, *Faecalicoccus*, *Gemmiger*, *Intestinibacter y Roseburia*

MS: Multiple Sclerosis; RRMS: Remittent Recurrent; PPMS: Primary Progressive; VS: versus; SCFAs: short chain fatty acids; AC: American Caucasian; AH: American Hispanic; AA: Afroamerican; α: Alpha; β: Beta; Diversity α: measures the variety of species present in a sample; Diversity β: measures differences in the composition of microbial communities between samples; ♂: men; ♀: women; +: Marginal; nc: non classified; *↓*: decrease in MS vs. control; *↑:* increase in MS vs. control.

**Table 3:** NOS risk of bias evaluation.

Reference	Selection	Comparability	Exposition	Conclusion
[17]	★★★★	★	★★	Low risk
[36]	★★★★	★	★★★	Low risk
[43]	★★★★	★	★★★	Low risk
[41]	★★★★	★	★★★	Low risk
[44]	★★★★	★	★★★	Low risk
[42]	★★★★	★	★★★	Low risk
[45]	★★★★	★	★★★	Low risk
[37]	★★★★	★	★★★	Low risk
[39]	★★★★	★	★★★	Low risk
[40]	★★★★	★	★★★	Low risk
[38]	★★★★	★	★★★	Low risk
[46]	★★★★	★	★★★	Low risk

★ indicates the quality of the studies, when the sum of the ★ is <4: low-quality study; 4–6 ★: moderate-quality study and ≥7 ★: high-quality study.

**Table 4:** Clue findings of relative abundances regarding taxon levels: MS vs. control group cases.

Taxon/Reference		[17]	[36]	[43]	[44]	[42]	[37]	[39]	[40]	[38]	[46]
Phylum	Firmicutes										
	Actinobacteria										
	Proteobacteria										
	Lentisphaerae										
	Bacteroidetes										
	Verrumicrobia										
Class	*Clostridio*										
Family	*Lachnospiraceae*										
	*Ruminococcaceae*										
Genre	*Bifidobacterium*										
	*Roseburia*										
	*Coprococcus*										
	*Butyricicoccus*										
	*Lachnospira*										
	*Akkermansia*										
	*Blautia*										
	*Parabacteroides*										
	*Dorea*										
	*Ruminococcus*										
	*Faecalibacterium*										
	*Prevotella*										
	*Methanobrevibacter*										
	*Dialister*										

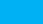
 Decrease in relative abundance MS/vs/Control, 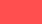 Increase in relative abundance MS/vs/Control.

## Data Availability

Not applicable.
